# The Safety and Efficacy of Endoscopic Combined Intrarenal Surgery (ECIRS) versus Percutaneous Nephrolithotomy (PCNL): A Systematic Review and Meta-Analysis

**DOI:** 10.1155/2022/1716554

**Published:** 2022-07-18

**Authors:** Victor A. Abdullatif, Roger L. Sur, Ziad A. Abdullatif, Sharon R. Szabo, Joel E. Abbott

**Affiliations:** ^1^Urology, Ascension Macomb-Oakland Hospital, Warren 48093, MI, USA; ^2^Urology, University of California San Diego Health, San Diego 92121, CA, USA; ^3^Research, Midwestern University College of Osteopathic Medicine, Glendale 85308, AZ, USA; ^4^Research, University of Michigan, Ann Arbor 48104, MI, USA; ^5^Clinical Research, Pacific West Urology, Las Vegas 89107, NV, USA

## Abstract

**Purpose:**

Our aim is to evaluate the safety and efficacy of endoscopic combined intrarenal surgery compared to percutaneous nephrolithotomy to guide practitioners and inform guidelines.

**Materials and Methods:**

A detailed database search was performed in PubMed, OVID, Scopus, and Web of Science in October 2021 to identify articles pertaining to ECIRS published between 2001 and 2021.

**Results:**

Four nonrandomized comparative studies and one RCT were identified, yielding five studies with a total of 546 patients (ECIRS/mini-ECIRS, *n* = 277; PCNL/mini-PCNL, *n* = 269). Subjects in these five studies met the predefined inclusion criteria established by two reviewers (J.E.A. and R.L.S.) and were therefore eligible for analysis. The results demonstrated that ECIRS was associated with a higher SFR (OR: 4.20; 95% CI: 2.79, 6.33; *p* < 0.00001), fewer complications (OR: 0.63; 95% CI: 0.41, 0.97; *p*=0.04), and a shorter hospital stay (WMD: −1.27; 95% CI: −1.55, −0.98; *p* < 0.00001) when compared to PCNL. There were no statistically significant differences in blood transfusions (OR: 0.45; 95% CI: 0.12, 1.68; *p*=0.24), operative time (SMD: −1.05; 95% CI: −2.42, 0.31; *p*=0.13), or blood loss (SMD: −1.10; 95% CI: −2.46, 0.26; *p*=0.11) between ECIRS and PCNL.

**Conclusions:**

ECIRS may be a more suitable approach for the surgical management of large and complex kidney stones currently indicating PCNL due to its superior efficacy with comparable surgical time and complication rate, though it is thought that a lack of resources and properly trained personnel may preclude ECIRS from becoming the standard. It is our impression that ECIRS may become the preferred technique in the endourologic community corresponding to the evolutionary sequence of percutaneous stone surgery.

## 1. Introduction

Percutaneous nephrolithotomy (PCNL) has been the standard treatment for upper- and midpole stones >20 mm and lower pole stones >10 mm for nearly 45 years [1, 2]. Although standard PCNL (24–30 Fr access sheath) has demonstrated the highest stone-free rate (SFR) of all stone-extraction procedures, caution is still exercised surrounding its use because it is the most invasive of the surgical options [3]. In consideration of the perioperative risks of standard PCNL, minimally invasive PCNL (mini-PCNL, 14–20 Fr access sheath) is now commonly used as an alternative [4]. Several systematic reviews and meta-analyses have demonstrated lower complication rates with mini-PCNL without compromising SFR [5, 6]. Although mini-PCNL has demonstrated improved safety compared to its predecessors, endoscopic combined intrarenal surgery (ECIRS) was developed with the goal of minimizing the number of access tracts of PCNL/mini-PCNL while simultaneously improving the one-step SFR.

Several nonrandomized comparative studies [7–14] (NRCSs) have reported conflicting evidence on the safety, efficiency, and efficacy of ECIRS compared to PCNL/mini-PCNL. Due to the lack of consensus on which of the two procedures is superior, this study seeks to determine the pooled effects of all studies that compare ECIRS/mini-ECIRS and PCNL/mini-PCNL in two interventional arms to guide practitioners and inform guidelines.

## 2. Materials and Methods

### 2.1. Research Question and PICO Model

This study aims to evaluate the safety and efficacy of ECIRS as compared to PCNL. The PICO model was used to define the research question as follows:

- Population: male and female patients diagnosed with single/multiple, large, complex, and/or high-burden renal stones in the upper urinary tract. All participants were eligible for treatment with PCNL or mini-PCNL in accordance with established urologic guidelines.
- Intervention: PCNL, ECIRS, mini-PCNL, and/or mini-ECIRS.
- Comparator: PCNL/mini-PCNL compared to ECIRS/mini-ECIRS matching for access sheath size.
- Outcomes: SFR, blood transfusion rate, complication rate, operative time, hospital stay, and blood loss.

### 2.2. Information Sources and Search Strategy

A search was performed in the following databases: PubMed, OVID, Scopus, and Web of Science. All databases were searched for relevant manuscripts from 2001 to 2021. The design for this systematic review was created using guidelines set forth by the Cochrane Handbook for Systematic Reviews of Interventions [15] and presented in the Preferred Reporting Items for Systematic Reviews and Meta-Analyses (PRISMA) statement [16]. In order to identify all studies pertaining to the research question, detailed electronic search strategies incorporating individual database-related search strategy modifications, such as filters, truncations, and wildcards, were developed and used in each electronic database. Boolean operators were used to generate both “free terms” and “index terms” that relate to ECIRS (e.g., “ECIRS”[term] OR “endoscopic combined intrarenal surgery”[term]). Details of the search terms and complete search queries used for each database are presented in the supplementary section under Appendix 1. Finally, reference list screening and citation tracking in Google Scholar were performed for each eligible article. This meta-analysis is exempt from Institutional Review Board approval as the data used in our manuscript is from previous clinical trials in which informed consent was already obtained by the individual trial researchers and investigators.

### 2.3. Inclusion Criteria and Exclusion Criteria

**Figure 1:**
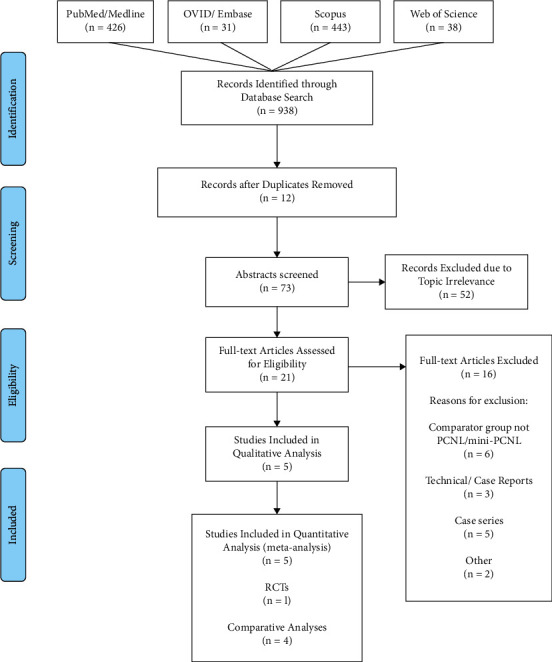
PRISMA flow diagram of the study selection process. PRISMA: Preferred Reporting Items for Systematic Reviews and Meta-Analyses [16].

Only randomized controlled trials (RCTs) or comparative studies that included patients receiving ECIRS (i.e., antegrade endoscopic stone surgery, combined with RURS/RIRS [1]) were included in our analysis. A particularly narrow range of study designs, that is, RCTs, quasi-RCTs—defined as RCTs in which allocation to treatment was obtained through a predictable method, such as with alternation, date of birth, or use of alternate medical records—and NRCSs, were deemed eligible for inclusion in our analysis. After identifying studies with one of the previous study designs, only studies that reported classical endourological parameters (i.e., stone-free rate) and surgical complications (bleeding, transfusions, and urosepsis) were included. Finally, only studies with parallel designs that compared an ECIRS/mini-ECIRS study arm and a PCNL/mini-PCNL study arm were included. Because both procedures include antegrade endoscopy as an obligatory component of the treatment modality, studies that did not match access sheath size in both groups were excluded. For example, if a study demonstrated inconsistency in tract diameter comparing mini-PCNL versus standard-ECIRS, or vice versa, it was excluded. Patients who underwent PCNL with multiple access tracts were initially deemed eligible for exclusion. However, upon review of the selected studies, only two patients in one study underwent mini-PCNL with two access tracts [17]. These patients were included in our analysis for several reasons. Firstly, the small sample size (*N* = 2) would have had a negligible effect on the pooled result. Secondly, the authors of this study did not indicate if these patients experienced different outcomes (SFR and complications), and therefore excluding them from the total may have precluded us from including the study. Two authors (J.E.A. and V.A.A.) independently determined that it would have been more valuable to include the study as it was the only RCT that met all the inclusion criteria. Studies were excluded from the analysis if the study design lacked a comparison between two study arms, as is the case with case/technical reports, reviews, conference papers, and retrospective/prospective case series. Details of the study selection process are presented in Figure 1. A list of all excluded studies with reasons for the exclusions is presented in the supplemental section under Appendix 4.

### 2.4. Types of Participants

The study participants included those diagnosed with single/multiple, large, complex, and/or high-burden renal stones in the upper urinary tract, where “large” and “complex” are defined as upper- and midpole stones >20 mm and lower pole stones >10 mm, and “high-burden” was defined as stones with an aggregate stone surface area >300 mm^2^, largest diameter >20 mm, or any branched stone (i.e., staghorn) occupying more than one portion of the renal collecting system. Patients with these stones were eligible for treatment with PCNL/mini-PCNL in accordance with either the American Urology Association [18] or European Association of Urology [19] guidelines for the surgical treatment of renal stones. No discrimination of sex, age, Body Mass Index (BMI), or American Society of Anesthesiologists (ASA) score was made by researchers and authors of the included studies.

### 2.5. Stone Characteristics

Four studies reported aggregate stone surface area (mm^2^) and/or largest diameter (mm) as means ± standard deviation, and one study [13] reported medians with corresponding ranges (lower bound (LB) to upper bound (UB)). In the study that reported stone diameter as a median (m(LBUB)), the means were determined as being equal to the median if the sample size of both arms was ≥70 [20]. The standard deviation was calculated with the following formula:

(1) $$\begin{array}{c} \sigma =\frac{R}{6}. \end{array}$$

Range (*R*) in (1) is equal to UB-LB [20]. Stone characteristics are summarized and presented in Table 1.

### 2.6. Types of Intervention

The procedures performed on eligible study participants included PCNL, ECIRS, mini-PCNL, and/or mini-ECIRS. Only studies that matched access sheath size in both groups were included to ensure that the alternative management strategy served as an appropriate comparator. Characteristics of these procedures reported by the authors included one or more of the following: patient position, number of urologists present, percutaneous access, type of nephroscope, type of ureteroscope, use/size of a ureteral access sheath, imaging technique used for puncture guidance (ultrasonographic *versus* endoscopic *versus* fluoroscopic), number of tracts, use/size of a postoperative JJ stent, use of a postoperative nephrostomy tube, and lithotripsy settings. These characteristics are presented in Tables 2 and 3, and additional details of operating techniques are presented in the supplementary materials under Appendix 2. In regard to lithotripsy, laser characteristics reported by the authors included one or more of the following: laser type, fiber size (*μ*m), pulse energy (Joules/pulse), and pulse frequency (Hz). Characteristics of lithotripsy are presented in Table 4. There was no discrimination for patient position (supine *versus* prone *versus* prone-modified *versus* supine-modified *versus* lateral decubitus *versus* GMSV) since it was consistent per study and based on surgeon preference.

### 2.7. Data Extraction and Analysis

Two authors (J.E.A. and V.A.A.) independently created spreadsheets with rows representing individual studies and columns representing the following variables: first author, publication year, study design/methodology, control/comparison group, sample size, gender, stone side, operative time, hospital stay, blood loss, blood transfusion rate, postoperative fever rate, SFR, and overall complication rate. Attempts were made to contact the original investigators if data was missing. Details of the emails sent to the authors are presented in the supplementary materials under Appendix 2. The quantitative degree of agreement between the reviewers' independent searches was calculated using the Cohen kappa statistic. A third author (R.L.S.) assessed the studies and data contained in both spreadsheets, and any disagreements were resolved by discussion.

### 2.8. Quality and Risk of Bias Assessment

Two authors (J.E.A. and V.A.A.) screened the titles, abstracts, methods, and results of all articles retrieved through the electronic search and obtained the full-text manuscripts for the meta-analysis. The methodologic quality and level of evidence of each study were independently evaluated by each reviewer. Criteria set forth by the Oxford Centre for Evidence-Based Medicine [21] were used to estimate the level of evidence in each study. In order to evaluate the risk of bias for studies with randomized protocols, the Cochrane Collaboration's risk of bias (RoB) tool [15] was used. The presence/absence of the following items was independently assessed by the reviewers: (1) adequate sequence generation; (2) adequate allocation concealment; (3) adequate removal of knowledge of the interventions from both participants/personnel and outcome assessors; (4) adequate appraisal of incomplete outcome data; (5) selective outcome reporting; (6) other variables that may induce a risk of bias (other bias). Using algorithms proposed by the risk of bias tool, we assigned each domain one of the following levels of bias:

- Low risk of bias (green symbol).
- Some concerns or unclear risk of bias (yellow symbol).
- High risk of bias (red symbol).

In order to assess the quality and risk of bias in studies with nonrandomized methodologies, the Risk of Bias in Nonrandomized Studies of Interventions (ROBINS-I) scale [22] was used. The ROBINS-I scale was used for the selected NRCSs, and the presence/absence of the following items—delineated as pre-, intra-, or postintervention domains—was assessed: (1) confounding variables; (2) exclusive inclusion of prevalent users, rather than new users, of the interventions (ECIRS, standard PCNL, and mini-PCNL); (3) differential or nondifferential misclassification of intervention status; (4) systematic differences between experimental interventions and comparator groups in the care provided; (5) missing data due to differential loss to follow-up or deliberate exclusion of individuals with missing information about intervention status or other variables; (6) differential or nondifferential errors in measurement of outcome; (7) selective reporting of results that prevents the estimate from being included in a meta-analysis. Using algorithms proposed by the ROBINS-I, a judgement was made on each domain with one of the following response options:

- “Yes,” in which there was an explicit presence of bias (purple circle).
- “Probably yes,” in which there was a likely presence of bias (red circle).
- “Probably no,” in which there was a moderate possibility of bias (yellow circle).
- “No,” in which there was an absence of bias (green circle).
- “No information” (blue circle).

### 2.9. Statistical Analysis

Data from each study was prepopulated into a spreadsheet and categorized as either dichotomous or continuous. Dichotomous variables included SFR, blood transfusion rate, and complication rate and are presented as proportions with corresponding odds ratios (ORs). Pooled estimates of ORs for studies that identified the previous dichotomous variables as clear data points were calculated. Continuous variables included operative time, hospital stay, and blood loss and are presented as means ± standard deviation with corresponding weighted or standardized mean differences (WMDs; SMDs). Pooled results are presented as weighted mean differences (WMDs) if units across all studies were the same or standard mean differences (SMDs) if units were different. Pooled estimates of WMDs/SMDs were calculated for studies that included continuous variables as clearly identifiable data points. The Mantel-Haenszel method was used to adjust the association between intervention (ECIRS/PCNL) and each dichotomous outcome for a potential, unobserved third variable [23]. Between-study heterogeneity was determined with Higgins *I*^*2*^, and the magnitude of heterogeneity was calculated with *χ*-square on N-1 degree of freedom with an alpha of 0.10. *I*^2^ values of 0% to 40%, 30% to 60%, 50% to 90%, and 90% to 100% correspond to minimal, moderate, substantial, and considerable levels of heterogeneity, respectively, and *I*^2^ values of ≤50% were regarded as acceptable.

To control for omitted variable bias, either a fixed-effects or random-effects model was used in our meta-analysis. The fixed-effects model was used if trials demonstrated minimal to low evidence of heterogeneity. A random-effects model was used if trials yielded heterogeneous results (*I*^2^ > 50%, *p* < 0.10). All levels of significance levels were set to a *p*-value of <0.05. Data from prepopulated spreadsheets were imported into Review Manager Version 5.4.1 (RevMan V.5.4, Cochrane Collaboration, Oxford, UK) to perform pairwise meta-analyses and display their accompanying forest plots and heterogeneity tests (*χ*^2^ and *I*^2^).

## 3. Results

### 3.1. Study Characteristics and Selection

Following a complete search of the literature with the inclusion and exclusion criteria, 21 full-text studies were identiﬁed, and a total of 5 studies [9, 10, 12, 13, 17] with a total of 546 patients (ECIRS/mini-ECIRS, *N* = 277; PCNL/mini-PCNL, *N* = 269) were included in the analysis. The Nuño de la Rosa study compared standard PCNL with standard-ECIRS [13], whereas the other 4 studies compared mini-PCNL with mini-ECIRS [9, 10, 12, 17].

### 3.2. Risk of Bias Results

**Figure 2:**
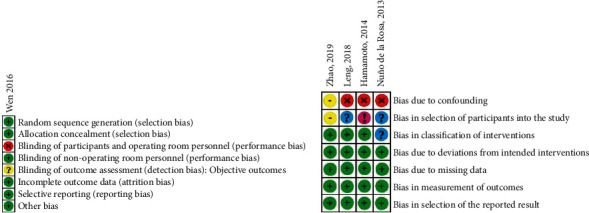
Risk of bias judgements (stone-free rate). Cochrane's RoB tool [15] for randomized studies (a); ROBINS-I tool [22] (b). Categories for Cochrane's RoB are “low risk,” “unclear risk,” and “high risk.” Green circle = low risk; yellow circle = unclear or equivocal risk; red circle = high risk. Categories for ROBINS-I are “low risk,” “moderate risk,” “serious risk,“ “critical risk,” and “no information.” Green circle = low risk; yellow circle = moderate risk; red circle = serious risk; pink circle = critical risk; blue circle = no information.

**Figure 3:**
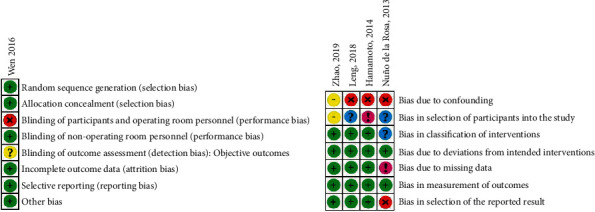
Risk of bias judgements (complications). Cochrane's RoB tool [15] for randomized studies (a) and ROBINS-I tool [22] (b). Categories for Cochrane's RoB are “low risk,” “unclear risk,” and “high risk.” Green circle = low risk; yellow circle = unclear or equivocal risk; red circle = high risk. Categories for ROBINS-I are “low risk,” “moderate risk,” “serious risk,” “critical risk,” and “no information.” Green circle = low risk; yellow circle = moderate risk; red circle = serious risk; pink circle = critical risk; blue circle = no information.

Risk of bias assessments were performed for both efficacy (SFR) and adverse events (complications). The results of the risk of bias assessments for both outcome measures are presented in Figures 2 and 3. For SFR, the overall risk of bias was low; therefore, firm conclusions on the efficacy of any reported treatment effects were able to be determined in our meta-analysis.

### 3.3. Dichotomous Outcomes

#### 3.3.1. Stone-Free Status

**Figure 4:**
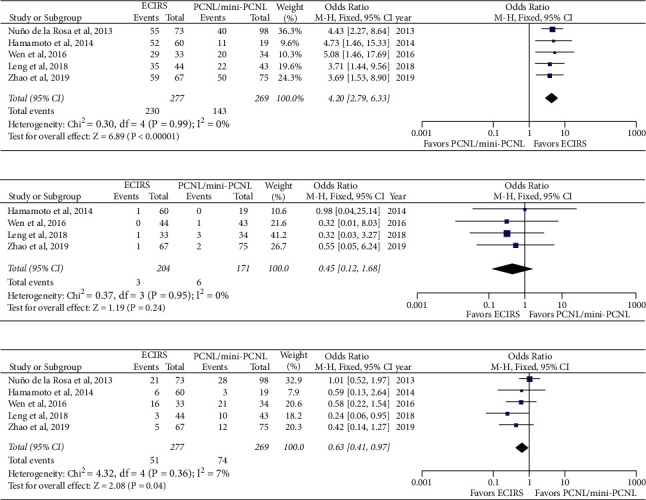
Forest plot of dichotomous outcomes between ECIRS and PCNL. Stone-free rate (a), blood transfusion rate (b), and complication rate (c). ECIRS: endoscopic combined intrarenal surgery; mini-PCNL: miniaturized percutaneous nephrolithotomy; PCNL: percutaneous nephrolithotomy; CI: confidence interval; M-H: Mantel-Haenszel [23].

All five studies reported stone-free status and were therefore included in the forest plot of dichotomous outcomes (Figure 4). With low heterogeneity among five studies (*I*^2^ = 0%, *p*=0.99), a fixed-effects model was applied to the calculation of differences in SFR and showed that ECIRS was associated with a higher SFR than PCNL (OR: 4.20; 95% CI: 2.79, 6.33; *p* < 0.00001) (Figure 4).

#### 3.3.2. Blood Transfusions

Four of the five studies provided data on blood transfusions in both the ECIRS and PCNL groups. Results of the meta-analysis by the fixed-effects model (*I*^2^ = 0%) demonstrated that there was no statistically significant difference in blood transfusion rate between ECIRS and PCNL (OR: 0.45; 95% CI: 0.12, 1.68; *p*=0.24) (Figure 4).

#### 3.3.3. Complications

All five studies provided data on complication rate. In cases where net complication rate was not explicitly provided, total complications were calculated as the sum of all reported Clavien–Dindo complications provided in the manuscript texts or tables. Results of the meta-analysis by the fixed-effects model demonstrated that ECIRS was associated with a lower complication rate than PCNL (OR: 0.63; 95% CI: 0.41, 0.97; *p*=0.04) (Figure 4).

### 3.4. Continuous Outcomes

#### 3.4.1. Operative Time

**Figure 5:**
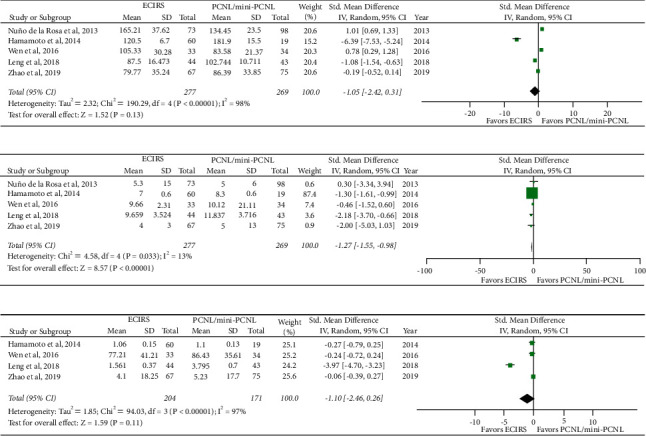
Forest plot of continuous outcomes between ECIRS and PCNL. Operative time (a), hospital stay (b), and blood loss (c). ECIRS: endoscopic combined intrarenal surgery; mini-PCNL: miniaturized percutaneous nephrolithotomy; PCNL: percutaneous nephrolithotomy; CI: confidence interval; M-H: Mantel-Haenszel [23].

Data on operative time (minutes) was provided by all five studies. Due to the variability in the definitions of operative time, a standardized mean difference (SMD) was used in lieu of a weighted mean difference (WMD). Pooled results demonstrated high levels of heterogeneity among the studies (*I*^2^ = 99%, *p* < 0.00001), and results of the random-effects model demonstrated no statistically significant difference in operative time between ECIRS and PCNL (SMD: −1.05; 95% CI: −2.42, 0.31; *p*=0.13) (Figure 5).

#### 3.4.2. Hospital Stay

All five studies provided data on hospital stay. Pooled results demonstrated low levels of heterogeneity (*I*^2^ = 13%, *p*=0.33). Results of the meta-analysis by the fixed-effects model demonstrated that ECIRS was associated with shorter hospital stay than PCNL (WMD: −1.27; 95% CI: −1.55, −0.98; *p* < 0.00001) (Figure 5).

#### 3.4.3. Blood Loss

Four of the five studies reported blood loss. Two studies [10, 12] reported blood loss as the drop in hemoglobin level using units of grams per deciliter (g/dL) for hemoglobin. Zhao et al. [9] reported blood loss as the drop in hemoglobin level using units of grams per liter (g/L) for hemoglobin. Lastly, Wen et al. [17] reported blood loss as the loss of total blood volume in milliliters (mL). Because of the variability in the units used to report blood loss across the four studies, an SMD was used in the analysis. With high levels of heterogeneity in the pooled results (*I*^2^ = 99%,  *p* < 0.00001), a random-effects model was used for our analysis. Results of the random-effects model showed no significant difference in blood loss between ECIRS and PCNL (SMD: −1.10; 95% CI: −2.46, 0.26; *p*=0.11) (Figure 5).

## 4. Discussion

To our knowledge, this is the first systematic review and meta-analysis to comprehensively analyze all comparative studies pertaining to ECIRS vis-à-vis PCNL/mini-PCNL. Based on the available evidence, it appears that the addition of RURS/RIRS to PCNL in patients with high stone burden is associated with higher SFR, lower overall complications, and shorter hospital stays than stand-alone PCNL/mini-PCNL with no significant differences in operative time, blood transfusion rates, and blood loss. This is of importance to the urologic community, providing an accessible and evidence-based rationale for a combined endoscopic approach to the treatment of stones that would otherwise be treated with percutaneous surgical techniques.

Only one systematic review has been published reporting data exclusively on ECIRS. Cracco and Scoffone [24] evaluated the efficacy of ECIRS by reporting ranges of outcome variables in all retrospective/prospective case series, NRCSs, and RCTs on ECIRS. However, no quantitative analyses were conducted. Although their review did not focus exclusively on PCNL as the comparator group, their findings were similar to ours, reporting that ECIRS was associated with higher SFRs. Interestingly, they also reported significant variability in the definition and calculation of operative time on ECIRS [24], which is in agreement with the significant heterogeneity in operative time in our meta-analysis (*I*^2^ = 99%).

The improved SFR that comes with the addition of retrograde ureteroscopy has been reported in the literature as early as 2003 [25]. Since then, ECIRS has demonstrated remarkable SFRs in multiple studies [26–28]. This is, in part, explained by the urologists' ability to perform stone removal with both a ureteroscope and a nephroscope, patently enhancing stone extraction and subsequently improving SFR. In our view, it is unsurprising that combining two stone-extraction procedures into one technique decreases a patient's likelihood of retaining occult stone fragments and therefore requiring a second procedure.

The efficacy of ECIRS, as measured by its improved SFR, is much less controversial than its effect on secondary outcomes such as overall complications, which is reported to be 5.8% to 42% [24]. PCNL monotherapy has a reported complication rate of 10.5% to 42% [24]. It is thought that the addition of RURS/RIRS to PCNL—as performed in ECIRS—compounds the risks of ECIRS by presenting an independent complication rate of 1.5%–12% [26–28]. In our estimation, the proposal of this theoretical increase in risk is problematic for several reasons. First, many of the probability estimates include both major and minor complications. The inclusion of minor complications, such as postoperative fever, ileus, wound infections, urinary tract infections (UTIs), and stent migration—although nontrivial—may lead to exaggerated estimations of risk. For example, Cracco and Scoffone have reported complication rates as low as 7.4% in a series of 310 patients after excluding minor complications [29]. For these reasons, it may be more statistically and clinically valuable to isolate complications by severity and discuss them in terms of absolute risk. In our view, extrapolating the estimates of overall complication rates to clinically significant data and patient-centric care is not practical or useful. Notwithstanding the issues involved in risk calculation, ECIRS was found to be associated with lower complications. A possible explanation for the lower rate of complications seen in ECIRS is the benefit ureteroscopy provides in allowing the operator to better visualize the intrarenal anatomy with two vantage points. It can aid in improving the precision of percutaneous renal access and also reducing the required antegrade torquing of the kidney as the surgeon works to visualize adjacent calyces to maximize stone clearance.

The other thought that may have prevented ECIRS from gaining widespread acceptance is the necessity of requiring two surgeons, therefore increasing cost. Although the majority of the included studies reported two surgeons for ECIRS, several authors have demonstrated the feasibility of performing ECIRS with a single surgeon. A recent analysis of 500 patients demonstrated safety and efficacy of ECIRS in a free-standing surgical center with only a single surgeon [30]. While the feasibility, safety, and efficacy of single-surgeon ECIRS have been demonstrated, we acknowledge that there may be a significant learning curve [31]. Even if two urologists are required and additional surgical equipment is needed, ECIRS is likely still providing a cost benefit to the healthcare system. Decreased surgical complications result in decreased cost, and higher stone-free rates result in fewer secondary procedures and fewer stone-related events, thus reducing cost. Because a stone nidus may result in stone growth, it is reasonable to assume that the improved SFRs of ECIRS would trickle down to decreased costs. The single-session SFR demonstrated in our pooled analysis substantiates this hypothesis of decreased cost. There were significantly more patients in the PCNL groups that required a second procedure. In our estimation, the reduced complication rate (including the decreased need for transfusions) and reduced risk for unplanned secondary interventions (with fewer second-look procedures [32]) outweigh the increased cost of having a second surgeon.

### 4.1. Limitations

Despite the rigor and thoroughness of our methods, we recognize a few limitations in our study. First, our pooled analysis included one randomized study and four nonrandomized studies potentially yielding a low level of evidence. This is due to the paucity of RCTs and comparative analyses on ECIRS versus PCNL. However, the included randomized study [12] was not significantly heterogeneous with respect to both primary and secondary outcomes, indicating that the pooled analysis was not significantly impacted by the RCT. Second, we recognize a potential limitation in the variable definitions of SFR. Although all of the selected studies included the absence of residual stone fragments as part of the “stone-free” definition, the authors defined “clinically insignificant” stone fragments with some variability. The authors of the included studies provided one of the following pairs of diagnostic methodologies and stone characteristics for their respective determinations of stone-free status: (1) postoperative CT scan after an unreported period of time to diagnose residual fragments, where any fragment <5 mm was considered clinically insignificant [13]; (2) plain abdominal radiography of the kidneys, ureters, and bladder (KUB), combined with renal ultrasonography (US) four weeks after surgery, where “stone free” was defined as the presence of no stones or only residual stone fragments of <4 mm in diameter [12]; (3) KUB and/or CT urography (CTU) one week after surgery where stones ≥4 mm in largest diameter were deemed clinically significant [17]; (4) KUB and CT urography (CTU) on postoperative day 1 or day 2 and 4 weeks after the surgery, where stone-free status was assigned with either no residual fragments or the largest diameter of fragments <4 mm [10]; (5) KUB or NCCT (for uncertain residual stone or radiolucent calculi) at four-week follow-up, where stone-free status was assigned in the absence of stones or presence of residual stone fragments <4 mm in diameter [9].

Moreover, one selected study did not use NCCT  scans for diagnosing stone-free status [12], the imaging modality with the highest established sensitivity for stones [19]. Despite the overall lack of strict definitions and diagnostics, we ascribed merit to the authors of the selected studies due to their use of identical diagnostic techniques in both the ECIRS and PCNL groups. Furthermore, our analysis demonstrated low heterogeneity in the pooled results indicating that any inconsistencies in the definitions of SFR were likely minor and therefore had little impact on the final results. Third, most of the included studies were nonrandomized comparative analyses for which there are inherent risks of confounding and selection bias. Fourth, publication bias among the five studies was found for complication rate. Finally, four of the five selected studies evaluated mini-PCNL, whereas one evaluated standard PCNL. Therefore, the generalizability of our data may have some limitations to standard PCNL. Although one may be concerned about the disparity between the selected studies, the tract sizes in said studies were all carefully matched across the study groups. Therefore, we deemed it appropriate to extrapolate our evidence to PCNL using any sized access sheath inasmuch as access sheath size is matched in a parallel fashion.

## Figures and Tables

**Table 1:** Stone size, multiplicity, and percutaneous access site.

Study	Stone size		Multiple stones, *N* (%)		Percutaneous puncture site, *N* (%)					
					Upper calyx		Middle calyx		Lower calyx	
	ECIRS	PCNL	ECIRS	PCNL	ECIRS	PCNL	ECIRS	PCNL	ECIRS	PCNL
Nuño de la Rosa et al. [13]	753.9 ± 343.4^*∗*^	649.5 ± 407.9^*∗*^	45 (46)	23 (39.8)	NS	NS	NS	NS	NS	NS
	39.9 ± 1.3	39.8 ± 1.1								
Hamamoto et al. [12]	Area NS	Area NS	33 (55)	6 (32)	NS	NS	NS	NS	NS	NS
	39.2 ± 2.6	38.4 ± 5.8								
Wen et al. [17]	689.23 ± 218.39^*∗*^	645.35 ± 232.51^*∗*^	NS	NS	20 (60.6)	18 (52.9)	12 (36.4)	15 (44.1)	25 (75.8)	22 (64.7)
	Length NS	Length NS								
Leng et al. [10]	Area NS	Area NS	NS	NS	NS	NS	NS	NS	NS	NS
	51.71 ± 9.42	52.77 ± 9.03								
Zhao et al. [9]	640.21 ± 377.22	753.44 ± 426.98^*∗*^	NS	NS	5 (41.7)	13 (43.3)	1 (8.3)	7 (23.3)	6 (50.0)	10 (33.3)
	Length NS	Length NS								

ECIRS: endoscopic intrarenal surgery; PCNL: percutaneous nephrolithotomy; NS: not specified. ^*∗*^mm2; all other units under stone size are in millimetres (mm).

**Table 2:** Characteristics of surgical design and devices used in the studies.

	Patient position		Urologists present		Percutaneous access sheath		Ureteroscope/nephroscope		Ureteral access sheath	
	ECIRS	PCNL	ECIRS	PCNL	ECIRS	PCNL	ECIRS	PCNL	ECIRS	PCNL
Nuño de la Rosa et al. [13]	GMSV	Supine	Two	NS	24/30 Fr	24/30 Fr	Karl Storz Flex X-2 and Olympus URF-P5	NS	11/13 Fr or 13/15 Fr	—
Hamamoto et al. [12]	Prone split-leg	Prone	Two	NS	18 Fr	18 Fr	Karl Storz Flex X-2	12 Fr miniscope	12/14 Fr	—
Wen et al. [17]	GMSV	Prone	Two	Two	20 Fr	20 Fr	Flexible ureteroscope	NS	12/14 Fr	—
Leng et al. [10]	Oblique supine lithotomic	Oblique supine lithotomic	NS	NS	16/18 Fr	16/18 Fr	Olympus URF-P5	NS	NS	—
Zhao et al. [9]	GMSV	Prone	Two	NS	16/18 Fr	16/18 Fr	7.5 Fr Flexible	NS	12/14 Fr	—

ECIRS: endoscopic intrarenal surgery; PCNL: percutaneous nephrolithotomy; GMSV: Galdakao-modified supine Valdivia (position). NS: not specified; Fr: French.

**Table 3:** Surgical characteristics similar for both procedures.

	Puncture guidance	Number of tracts	Postoperative ureteral stent	Postoperative nephrostomy
Nuño de la Rosa et al. [13]	NS	One	NS	NS
Hamamoto et al. [12]	US + fluoro	One	4.7 Fr	18 Fr
Wen et al. [17]	US	One	6 Fr	16 Fr
Leng et al. [10]	US (with Doppler)	One	5/7 Fr	16 Fr
Zhao et al. [9]	US + endoscopic	One	6 Fr	NS

NS: not specified; Fr: French; US: ultrasound; Fluoro: fluoroscopy.

**Table 4:** Laser characteristics in the included studies.

	Type		Fiber size (*μ*m)		Pulse energy (joules/pulse)		Frequency (Hz)	
	ECIRS	PCNL	ECIRS	PCNL	ECIRS	PCNL	ECIRS	PCNL
Nuño de la Rosa et al. [13]	NS	NS	NS	NS	NS	NS	NS	NS
Hamamoto et al. [12]	Ho:YAG	Ho:YAG	200 or 365	200 or 365	NS	NS	NS	NS
Wen et al. [17]	Ho:YAG	Ho:YAG	200	550	0.8–1.5	1.0–1.5	15–30	15–20
Leng et al. [10]	Ho:YAG	Ho:YAG	200	200	0.8–1.2	0.8–1.2	10–20	10–20
Zhao et al. [9]	Ho:YAG	Ho:YAG	550	550	1.5–3.0	1.5–3.0	20–30	20–30

Ho:YAG: holmium-yttrium-aluminum-garnet; ECIRS: endoscopic combined intrarenal surgery; mini-PCNL: miniaturized percutaneous nephrolithotomy; NS: not specified.

## Data Availability

The data used to support the findings of this study are included within the supplementary information file.

## Supplementary Materials

Supplementary Materials The Supplemental Materials contains four appendices, which are as follows. *Appendix 1.* Search queries used for electronic search strategy during systematic review. *Appendix 2.* Main characteristics of populations, interventions, and outcome measures of included randomized trials and nonrandomized comparative studies. *Appendix 3.* Risk of bias results using ROBINS-I for nonrandomized studies and Cochrane's Risk of Bias 2.0 for randomized studies. *Appendix 4.* Characteristics of excluded studies with justifications for exclusion.
