# Supplementary material for: The Safety and Efficacy of Endoscopic Combined Intrarenal Surgery (ECIRS) versus Percutaneous Nephrolithotomy (PCNL): A Systematic Review and Meta-Analysis
Source: Adv Urol. 2022 Jul 18;2022:1716554. doi: 10.1155/2022/1716554 (PMC9314160; doi:10.1155/2022/1716554)
Supplement: Supplementary Materials — The Supplemental Materials contains four appendices, which are as follows. Appendix 1. Search queries used for electronic search strategy during systematic review. Appendix 2. Main characteristics of populations, interventions, and outcome measures of included randomized trials and nonrandomized comparative studies. Appendix 3. Risk of bias results using ROBINS-I for nonrandomized studies and Cochrane's Risk of Bias 2.0 for randomized studies. Appendix 4. Characteristics of excluded studies with justifications for exclusion. [file 1716554.f1.docx]

# **Supplemental Materials**

## **Appendix 1**. Search queries used for electronic search strategy during systematic review.

**PubMed/Medline**

For searches conducted in the PubMed database, the following terms were used on 10/28/2021 at 16:45:00:

("PCNL"[All Fields] OR ("nephrolithotomy, percutaneous"[MeSH Terms] OR ("nephrolithotomy"[All Fields] AND "percutaneous"[All Fields]) OR "percutaneous nephrolithotomy"[All Fields] OR ("percutaneous"[All Fields] AND "nephrolithotomy"[All Fields])) OR (("percutaneous"[All Fields] OR "percutaneously"[All Fields] OR "percutanous"[All Fields]) AND "nephrolithotripsy"[All Fields]) OR "PNL"[All Fields] OR ("micropercutaneous"[All Fields] AND ("nephrolithotomies"[All Fields] OR "nephrolithotomy"[All Fields])) OR ("micropercutaneous"[All Fields] AND "nephrolithotripsy"[All Fields]) OR "mini-PCNL"[All Fields] OR (("miniature"[All Fields] OR "miniatures"[All Fields] OR "miniaturization"[MeSH Terms] OR "miniaturization"[All Fields] OR "miniaturizations"[All Fields] OR "miniaturize"[All Fields] OR "miniaturized"[All Fields] OR "miniaturizes"[All Fields] OR "miniaturizing"[All Fields]) AND ("nephrolithotomy, percutaneous"[MeSH Terms] OR ("nephrolithotomy"[All Fields] AND "percutaneous"[All Fields]) OR "percutaneous nephrolithotomy"[All Fields] OR ("percutaneous"[All Fields] AND "nephrolithotomy"[All Fields])))) AND ((("combinable"[All Fields] OR "combinated"[All Fields] OR "combination"[All Fields] OR "combinational"[All Fields] OR "combinations"[All Fields] OR "combinative"[All Fields] OR "combine"[All Fields] OR "combined"[All Fields] OR "combines"[All Fields] OR "combining"[All Fields]) AND ("intrarenal"[All Fields] OR "intrarenally"[All Fields]) AND ("surgery"[MeSH Subheading] OR "surgery"[All Fields] OR "surgical procedures, operative"[MeSH Terms] OR ("surgical"[All Fields] AND "procedures"[All Fields] AND "operative"[All Fields]) OR "operative surgical procedures"[All Fields] OR "general surgery"[MeSH Terms] OR ("general"[All Fields] AND "surgery"[All Fields]) OR "general surgery"[All Fields] OR "surgery s"[All Fields] OR "surgerys"[All Fields] OR "surgeries"[All Fields])) OR "ECIRS"[All Fields] OR (("flexibilities"[All Fields] OR "flexible"[All Fields] OR "flexibles"[All Fields] OR "pliability"[MeSH Terms] OR "pliability"[All Fields] OR "flexibility"[All Fields]) AND ("nephroscopies"[All Fields] OR "nephroscopy"[All Fields])) OR (("flexibilities"[All Fields] OR "flexible"[All Fields] OR "flexibles"[All Fields] OR "pliability"[MeSH Terms] OR "pliability"[All Fields] OR "flexibility"[All Fields]) AND ("ureteroscopy"[MeSH Terms] OR "ureteroscopy"[All Fields] OR "ureteroscopies"[All Fields])) OR ("Galdakao-modified"[All Fields] AND ("supinate"[All Fields] OR "supinated"[All Fields] OR "supinates"[All Fields] OR "supinating"[All Fields] OR "supination"[MeSH Terms] OR "supination"[All Fields] OR "supinations"[All Fields] OR "supinator"[All Fields] OR "supinators"[All Fields] OR "supine position"[MeSH Terms] OR ("supine"[All Fields] AND "position"[All Fields]) OR "supine position"[All Fields] OR "supine"[All Fields]) AND "Valdivia"[All Fields] AND ("patient positioning"[MeSH Terms] OR ("patient"[All Fields] AND "positioning"[All Fields]) OR "patient positioning"[All Fields] OR "positioning"[All Fields] OR "position"[All Fields] OR "position s"[All Fields] OR "positional"[All Fields] OR "positioned"[All Fields] OR "positionings"[All Fields] OR "positions"[All Fields])))

References retrieved: 426

**OVID / Cochrane Library and Embase**

For searches conducted in the OVID (Cochrane Library and Embase) database, the following terms were used on 10/28/2021 at 17:32:00:

((' PCNL '/exp OR ' nephrolithotomy, percutaneous OR ' nephrolithotomy ' OR ' percutaneous ' OR ' percutaneous nephrolithotomy OR micropercutaneous ' PCNL OR percutaneous nephrolithotomy OR (percutaneous nephrolithotripsy OR PNLOR micropercutaneous nephrolithotomy OR micropercutaneous nephrolithotripsy OR (mini-PCNL)) OR (miniaturized percutaneous nephrolithotomy AND combined intrarenal surgery OR (ECIRS OR flexible nephroscopy OR flexible ureteroscopy OR Galdakao-modified supine Valdivia position)

References retrieved: 31

**Scopus**

For searches conducted in the Scopus database, the following terms were used 10/28/2021 at 18:14:00:

PCNL* OR “percutaneous nephrolithotomy” OR “percutaneous nephrolithotripsy” OR “PNL” OR “micropercutaneous nephrolithotomy” OR “micropercutaneous nephrolithotripsy” OR “mini-PCNL” OR “miniaturized percutaneous nephrolithotomy” AND combined intrarenal surgery* OR “ECIRS” OR “flexible nephroscopy” OR “flexible ureteroscopy” OR “Galdakao-modified supine Valdivia position”

References retrieved: 443

**Web of Science**

For searches conducted in the Web of Science database, the following terms were used on 10/28/2021 at 18:26:00:

TS=(PCNL) OR (percutaneous nephrolithotomy) OR (percutaneous nephrolithotripsy) OR (PNL) OR (micropercutaneous nephrolithotomy)OR (micropercutaneous nephrolithotripsy) OR (mini-PCNL) OR (miniaturized percutaneous nephrolithotomy)) AND TS=(combined intrarenal surgery) OR (ECIRS) OR (flexible nephroscopy)) OR (flexible ureteroscopy) OR (Galdakao-modified supine Valdivia position)

References retrieved: 38

| **Nuño de la Rosa et al. 2013** | |
| --- | --- |
| **Methods** | **Study design:** Nonrandomized comparative study  **Study period:** January 2005 to December 2011 |
| **Participants** | **Setting:** Inpatient  **Country:** Spain  **Population:** Both males and females who underwent ECIRS or standard (“classical”) PCNL. Groups were matched for previous stone surgeries and age.  **Number:** ECIRS group (*N* = 73); standard PCNL group (*N* = 98); Total (*N* = 171)  **Mean age:** ECIRS group (52.6 ± 1.7); Standard PCNL group (50.5 ± 1.3) |
| **Interventions** | **ECIRS group**   - Patient position: Galdakao-Modified Supine Valdivia (GMSV) - Urologists present: Two - Percutaneous access: Amplatz 24—30 Fr - Ureteroscope: Karl-Storz Flex X2 and URF-P5 Olympus - Ureteral access sheath: 11/13 Fr or 13/15 Fr - Puncture guidance: Not specified - Number of tracts: One   - Cases performed with multiple access sites were excluded from the series - Lithotripsy characteristics: Not specified - Postoperative JJ stent: Not specified - Postoperative nephrostomy tube: Not specified   **Standard PCNL group**   - Patient position: Supine - Urologists present: Not specified - Percutaneous access: Amplatz 24—30 Fr - Cystoscope: Karl-Storz 17 Fr flexible cystoscope - Nephroscope: Not specified - Ureteral access sheath: Not applicable - Puncture guidance: Not specified - Number of tracts: One   - Cases performed with multiple access sites were excluded from the series - Lithotripsy characteristics: Not specified - Postoperative JJ stent: Not specified - Postoperative nephrostomy tube: Not specified |
| **Outcomes** | **Primary outcome(s):**   - Stone-free rate   - Defined as the quote, “absence of lithiasis or with residual calculi smaller than 5 mm.”   - Quote: “Postoperative CT was performed in most patients to evaluate results, except in speciﬁc cases where standard plain ﬁlm radiography was used (exclusively in cases of smaller calculi; cavity molds). The PCNL procedure was considered successful when there was absence of lithiasis or with residual calculi smaller than 5 mm. The overall success of the treatment was also assessed, deﬁning this as the sum of the PCNL results and a second-line treatment, when the patient achieves stone-free status or has non-signiﬁcant residual calculi. A second-line treatment was indicated in those patients with residual calculi larger than 7 mm, since spontaneous expulsion was considered unlikely. The percentage of eliminated stones was deﬁned as the percentage of eliminated stones after the ﬁrst procedure, in terms of stone surface area.”   **Secondary outcome(s):**   - Adverse events   - Complications     - The authors only reported overall complication rate with no indication as to what was included in the term *complications.*     - Quote: “A modiﬁed Clavien grading system [6] was used to classify the complications.” - Perioperative variables/parameters   - Days of hospital stay |
| **Funding sources** | None reported |
| **Declarations of interest** | None |
| **Notes** | Language of publication: Spanish (original); translated to English  Email to the authors sent on 10/29/2021:   - “We are interested in the following:   - Specifics of the ‘complications’ data: Did you happen to stratify ‘complications’ by type and severity?   - Do you have the total number of patients that experienced said complications stratified by severity/Clavien-Dindo classification?”   - What are the details of the lithotripsy used in the stone extirpation portion of both procedures?” |

## **Appendix 2.** Main Characteristics of Populations, Interventions, and Outcome Measures of Included Randomized Trials and Nonrandomized Comparative Studies

| **Hamamoto et al. 2014** | |
| --- | --- |
| **Methods** | **Study design:** Nonrandomized comparative study  **Study period:** February 2004 to January 2013 |
| **Participants** | **Setting:** Inpatient  **Country:** Japan  **Population:** Quote: “All those who were candidates for PCNL surgery as the primary indication based on the EAU and American Urological Association (AUA) guidelines were eligible for the study. Inclusion criteria were single or multiple renal stones including staghorn stones, stone diameter > 2 cm, and no contraindications to perform PCNL surgery in the prone position. There were no speciﬁc exclusion criteria. Treatment of patients with PCNL was based on the presence of symptoms of pain in the ﬂank, hematuria, fever, and/or sepsis.  **Number:** ECIRS (N = 60); mini-PCNL (N = 19); total (N = 79)  **Mean age:** ECIRS (54.5 ± 1.5); mini-PCNL (48.9 ± 3.3) |
| **Interventions** | **ECIRS group**   - Patient position: Prone split-leg - Urologists present: Two - Percutaneous access: 18 Fr - Ureteroscope: Karl-Storz Flex X2 - Ureteral access sheath: 12/14 Fr - Puncture guidance: Ultrasound and fluoroscopy - Number of tracts: One - Lithotripsy characteristics:   - Quote: “A 200- or 365-μm YAG laser ﬁber was used in conjunction with fURS to fragment the renal calculi.” - Postoperative JJ stent: 4.7 Fr - Postoperative nephrostomy tube:  18 Fr   **mini-PCNL group**   - Patient position: Prone - Urologists present: Not specified - Percutaneous access: 18 Fr - Ureteroscope: Not applicable - Nephroscope: 12 Fr miniscope (Karl Storz) - Ureteral access sheath: Not applicable - Puncture guidance: Ultrasound and fluoroscopy - Number of tracts: One - Lithotripsy characteristics:   - Quote: “Stone disintegration was performed using a lithoclast or holmium- yttrium-aluminum-garnet (YAG) laser (Boston Scientiﬁc Japan K.K. Tokyo, Japan)” - Postoperative JJ stent: Not specified - Postoperative nephrostomy tube: 18 Fr |
| **Outcomes** | **Primary outcome(s):**   - Stone-free rate   - Defined as quote, “no stones or only residual stone fragments of <4 mm in diameter.”   - Quote: "The SFR was determined 4 weeks after surgery by plain abdominal radiography of the kidneys, ureters, and bladder and renal sonography. 'Stone free' was deﬁned as presence of no stones or only residual stone fragments of <4 mm in diameter."   **Secondary outcome(s):**   - Adverse events / complications   - Blood transfusion     - Defined as any patient that required a blood transfusion   - Fever     - Defined as a body temperature > 38.5 °C (101.3 °F)   - Quote: “Perioperative complications were graded according to the modiﬁed Clavien classiﬁcation system as applied to PCNL. [9,17]” - Perioperative variables/parameters   - Total length operation (minutes)     - Authors defined total length operation as, quote, “surgery time, including time for patient positioning”   - Blood loss     - Authors reported blood loss as the drop in hemoglobin (g/dL) and hematocrit (%)   - eGFR drop     - Measured in ml/min/1.73m^2^   - Hospital stay     - Defined as the number of days patients remained in the hospital postoperatively   - Number of ancillary treatments     - Ancillary treatments included second PCNL, shockwave lithotripsy (SWL), ureteroscopy (URS), or “mixed” in which a combination of two aforementioned treatments were performed |
| **Funding sources** | None reported |
| **Declarations of interest** | None |
| **Notes** | Language of publication: English |

| **Wen et al. 2016** | |
| --- | --- |
| **Methods** | **Study design:** Randomized controlled trial (RCT)  **Period of surgery:** May 2012 to October 2014 |
| **Participants** | **Setting:** Inpatient  **Country:** China  **Population:** Patients over 18 years old diagnosed with partial staghorn calculi. Inclusion criteria were patients with partial staghorn calculi detected by CTU and plain KUB. Exclusion criteria were patients with renal anatomic anomalies, coagulation disorders, previous PCNL history, solitary kidney, severe urinary infection or tuberculosis, and severe cardiac and pulmonary dysfunction.  **Number:** ECIRS group (N = 33); mini-PCNL group (N = 34); total (N = 67)  **Mean age:** ECIRS group (43.18 ± 14.11); mini-PCNL group (45.76 ± 13.25) |
| **Interventions** | **ECIRS group**   - Patient position: Galdakao-Modified Supine Valdivia (GMSV) - Percutaneous access: 20 Fr - Ureteroscope: Flexible ureteroscope (size/brand not specified) - Ureteral access sheath: 12/14 Fr - Puncture guidance: Ultrasound - Number of tracts: One - Lithotripsy characteristics:   - Quote: “The stones were fragmented with Ho:YAG laser equipped with 200μm fiber. The parameters of Ho:YAG laser energy were set with 0.8-1.5 J at frequency of 15-30 Hz. Stone fragments were evacuated by basket or removed through MPCNL tract under nephroscopy.” - Postoperative JJ stent: 6 Fr - Postoperative nephrostomy tube:  16 Fr*   - Quote: “After the lithotripsy and remove of the stone fragments, a 6F double J stent (Cook Medical, Indiana, USA) was placed and a 16F nephrostomy tube was routinely inserted.”   - Authors did not explicitly state that they used a 16 Fr nephrostomy tube in the ECIRS methods section. This was assumed based on quote “The MPCNL surgical procedure *was described as above*, and the retrograde intrarenal surgery was applied simultaneously.”   **mini-PCNL group**   - Patient position: Prone - Percutaneous access: 20 Fr - Ureteroscope: Not applicable - Nephroscope: Not specified - Ureteral access sheath: Not applicable - Puncture guidance: Ultrasound - Number of tracts: One - Lithotripsy characteristics:   - Quote: “The stones were fragmented with Ho:YAG laser with 550μm fiber (Power Suite 100W Plus, Lumenis) under nephroscope. The energy of Ho:YAG laser ranged from 1.0 J to 1.5 J and the frequency ranged from 15 Hz to 20 Hz based on the stone rigidity” - Postoperative JJ stent: 6 Fr - Postoperative nephrostomy tube: 16 Fr |
| **Outcomes** | **Primary outcome(s):**   - Stone-free rate   - Quote: “The stone free status was defined as residual fragments <4 mm.”   - Authors performed KUB and/or CTU at one month follow-up to determine stone-free status.   **Secondary outcome(s):**   - Adverse events / complications   - Transient fever     - Defined as a body temperature > 38.5 °C (101.3 °F)   - Urosepsis     - Quote: “…3 urosepsis (two due to *Escherichia coli*, another one due to *Enterococcus faecalis*) after the operation, which was responsive to anti-infective treatment according to urine culture results (Modiﬁed Clavien Classiﬁcation, grade 2)”   - Urinary leakage     - No definition provided   - Significant hemorrhage requiring blood transfusion     - No definition for what constituted “significant” hemorrhage. Authors mention that mean blood loss for patients requiring transfusion was 510 ml   - Arteriovenous fistula in puncture site     - Quote: “Arteriovenous ﬁstula in the puncture site was detected by digital subtraction angiography in 1 patient, who underwent blood transfusion and embolisation (grade 3).” - Perioperative variables/parameters   - Operative time (minutes)     - No explicit definition for operative time provided or descriptions of how it was determined   - Blood loss     - Authors determined blood loss by measuring the mass of hemoglobin in the intraoperative irrigation fluid and urine and reported it in milliliters (ml).   - Number of percutaneous access tracts   - Hospitalization time (days) |
| **Funding sources** | ﻿﻿﻿Funding was provided by grants from National Natural Science Foundation of China (No. 81500532 to Bohan Wang and No. 81300475 to Jiaming Wen) |
| **Declarations of interest** | None |
| **Notes** | Language of publication: English  Email to the authors:  “In table 2, we noticed that 2 patients required 2 percutaneous access tracts. We are particularly interested in knowing if those two patients were the one who had complications, had different outcomes, etc. Do you have this data available? Secondly, which computer-generated allocation sequence was used for randomization?” |

| **Leng et al. 2018** | |
| --- | --- |
| **Methods** | **Study design:** Nonrandomized comparative study  **Study duration:** March 2014 to January 2016 |
| **Participants** | **Setting:** Inpatient  **Country:** China  **Population:** Patients with staghorn calculi (SC) diagnosed via kidney-ureter-bladder (KUB) plain image, ultrasound, intravenous urography, and/or computed tomography (CT) scan. All patients had unilateral SC.  **Number:** ECIRS group (N = 44); mini-PCNL group (N = 43); total (N = 87)  **Mean age:** ECIRS group (46.182 ± 12.743); mini-PCNL group (45.767 ± 11.223) |
| **Interventions** | **ECIRS group**   - Patient position: Oblique supine lithotomic - Urologists present: Two - Percutaneous access: 16 or 18 Fr - Ureteroscope: URF-P5 Olympus - Ureteral access sheath: Authors mentioned the use of a ureteral access sheath in the discussion. They did not specify the size/brand or how it was implemented in the methods - Puncture guidance: Doppler ultrasound - Number of tracts: One - Lithotripsy characteristics:   - Quote: “A holmium laser was used at an energy setting of 0.8 to 1.2 J and a frequency of 10 to 20 Hz. The size of the laser fiber was 200 µm. Stone fragments were removed through the percutaneous renal access after the residual stones were crushed.” - Postoperative JJ stent: 5/7 Fr - Postoperative nephrostomy tube: 16 Fr   **PCNL group**   - Patient position: Oblique supine lithotomic - Urologists present: Not specified - Percutaneous access: 16 or 18 Fr - Ureteroscope: Not applicable - Nephroscope: Not specified - Ureteral access sheath: Not applicable - Puncture guidance: Doppler ultrasound - Number of tracts: One - Lithotripsy characteristics:   - Quote: “A holmium laser was used at an energy setting of 0.8 to 1.2 J and a frequency of 10 to 20 Hz. The size of the laser fiber was 200 µm. Stone fragments were removed through the percutaneous renal access after the residual stones were crushed.” - Postoperative JJ stent: 5/7 Fr - Postoperative nephrostomy tube: 16 Fr |
| **Outcomes** | **Primary outcome(s):**   - Stone-free rate   - Quote: “The SFR was defined as no residual fragments, or the diameter of fragments were less than 4 mm by computed tomography and kidney-ureter-bladder plain image…”   - Authors performed KUB and CT postoperative day 1 or day 2, and 4 weeks after the surgery to determine stone-free status.   **Secondary outcome(s):**   - Adverse events / complications   - Postoperative fever     - Defined as a body temperature ≥ 38.5 °C (101.3 °F)   - Postoperative blood transfusion   - Postoperative pleural injury - Perioperative variables   - Operative time (minutes)     - No explicit definition for operative time provided or descriptions of how it was determined   - Hospitalization time (days)   - Blood loss     - Authors reported blood loss as the drop in hemoglobin (g/dL) |
| **Funding sources** | Funding was provided by grants from the Gannan Medicine University Postgraduate Innovation Fund, Jiangxi, China (YC2015-X 008) |
| **Declarations of interest** | None |
| **Notes** | Language of publication: English |

| **Zhao et al. 2019** | |
| --- | --- |
| **Methods** | **Study design:** Nonrandomized comparative study  **Study duration:** January 2005 to December 2011 |
| **Participants** | **Setting:** Inpatient  **Country:** China  **Population:** Patients diagnosed with multiple pyelocaliceal stones or staghorn renal calculi.  **Number: ﻿**ECIRS (N = 66); mini-PCNL (N = 74); total (N = 140)  **Mean age:** ECIRS (53.18 ± 12.66); mini-PCNL (53.10 ± 13.18) |
| **Interventions** | **ECIRS group**   - Patient position: Galdakao-Modified Supine Valdivia (GMSV) - Urologists present: Two - Percutaneous access: 16/18 Fr - Ureteroscope: 7.5 Fr flexible - Ureteral access sheath: 12/14 Fr - Puncture guidance: Ultrasound / endoscope - Number of tracts: One - Lithotripsy characteristics:   - Quote: “The main parts of the calculi were fragmented with a 550 μm holmium laser fiber with energy of 1.5 to 3.0 J at 20 to 30 Hz through the percutaneous tract. And then, the residual stones were further dusted with a 200 μm holmium laser fiber and 0.5 to 1.0 J of energy at a frequency of 30 to 40 Hz through the retrograde approach. Nitinol stone baskets or stone forceps under flexible ureteroscopy were used if necessary.” - Postoperative JJ stent: 6 Fr - Postoperative nephrostomy tube: Authors state that a nephrostomy tube was placed, but did not indicate the size   **PCNL group**   - Patient position: Prone - Urologists present: Not specified - Percutaneous access: 16/18 Fr - Ureteroscope: Not applicable - Nephroscope: Not specified - Ureteral access sheath: Not applicable - Puncture guidance: Ultrasound - Number of tracts: One - Lithotripsy characteristics:   - Quote: “Except for the usage of flexible instrumentation, the rest of the steps was same as mentioned above.” - Postoperative JJ stent: 6 Fr*   - Authors did not explicitly state that they used a 6 Fr JJ in the MPCNL methods section. This was assumed based on quote, "Except for the usage of flexible instrumentation, the rest of the steps was same as mentioned above." - Postoperative nephrostomy tube: Authors state that a nephrostomy tube was placed, but did not indicate the size   - Authors did not explicitly state that they used a postoperative nephrostomy in the MPCNL methods section. This was assumed based on quote, "Except for the usage of flexible instrumentation, the rest of the steps was same as mentioned above." |
| **Outcomes** | **Primary outcome(s):**   - Stone-free rate   - Defined as, quote, “…absence of stone or residual stone fragments < 4 mm in diameter (clinically insignificant residual fragments).”   - Authors determined stone-free status by KUB or NCCT (for radiolucent/uncertain calculi) at 4-week follow-up.   **Secondary outcome(s):**   - Adverse events / complications   - Postoperative fever     - Defined as a body temperature > 38.5 °C (101.3 °F)   - Postoperative blood transfusion   - Postoperative pleural injury - Perioperative variables   - Operative duration (minutes)     - Authors defined operative duration as the time from positioning to the end of the procedure.   - Postoperative hospitalization (days)   - Blood loss     - Authors reported blood loss as the drop in hemoglobin (g/dL)   - Changes in serum creatinine (SCr)     - Reported in ﻿﻿μmol/L |
| **Funding sources** | Funding was provided by Beijing Municipal Administration of Hospitals Clinical medicine Development of special funding support, code: XMLX201826. |
| **Declarations of interest** | None |
| **Notes** | Language of publication: English |

## **Appendix 3.** Risk of Bias Results Using ROBINS-I for Nonrandomized Studies and Cochrane’s Risk of Bias 2.0 for Randomized Studies

| **Nuño de la Rosa et al. 2013** | | |
| --- | --- | --- |
| **Pre-intervention bias** | **Authors’ judgement** | **Support for judgement** |
| Bias due to confounding | Serious | Adjustment was only made for stone characteristics. No adjustment was made for comorbidities. |
| Bias in selection of participants into the study | Unclear | ﻿﻿This is a retrospective study comparing two surgical techniques (PCNL versus standard ECIRS). ECIRS may have been used in patients with a higher stone burden due to the complementary addition of retrograde ureteroscopy. Case selection may therefore have influenced the estimate of the treatment effect in the ECIRS arm. It was unclear how participants were selected for treatment with ECIRS over PCNL. |
| **At intervention** | **Authors’ judgement** | **Support for judgement** |
| Bias in classification of interventions | Unclear | ECIRS and mini-PCNL groups are incompletely defined. Characteristics of lithotripsy not included in description of surgical technique.  ﻿﻿﻿Quote: "All patients underwent PCNL in the supine position, which was performed at a hospital in Galdakao. We divided the series into 2 subgroups, PCNLs (subgroup where no ﬂexible endoscopes were used), ECIRS (subgroup where a nephroscope/ﬂexible ureteroscope was used). In the ECIRS subgroup, the ureteral access sheath was always inserted (Navigator 11/13 or 13/15F BostonScientiﬁc®, ReTrace 12/14F Coloplast®) and the Karl-Storz17F ﬂexible cystoscope and the Karl-Storz Flex X2 and Olympus P5 ﬂexible uretero-scopes were used as ﬂexible endoscopes. All the procedures included in this study were performed through a single per-cutaneous access and with an Amplatz 24---30 F sheath (those cases performed with multiple access sites were excluded from the series)." |
| **Post-intervention bias** | **Authors’ judgement** | **Support for judgement** |
| Bias due to deviations from intended interventions | Low | All participants received intended intervention as described in surgical methods. |
| Bias due to missing data | Low (stone-free rate)  Critical (complications) | Data on stone-free rate were reasonably complete﻿  No data on complications experienced by both groups available |
| Bias in measurement of outcomes | Low (SFR)  Low (complications) | Blinding of assessors, ﻿personnel, or participants was not applied. However, we concluded on scientific judgement that the occurrence of events was unrelated to blinding. |
| Bias in selection of the reported results | Low (SFR)  Serious (complications) | Authors only reported complication rate without delineation of complications |

| **Hamamoto et al. 2014** | | |
| --- | --- | --- |
| **Pre-intervention bias** | **Authors’ judgement** | **Support for judgement** |
| Bias due to confounding | Serious | No adjustment was made for comorbidities or previous treatments. |
| Bias in selection of participants into the study | Critical | Small sample size in mini-PCNL group (*N* = 19) compared to mini-ECIRS group (*N* = 60). Unclear how participants were selected for mini-ECIRS over mini-PCNL. |
| **At intervention** | **Authors’ judgement** | **Support for judgement** |
| Bias in classification of interventions | Low | Classification of interventions clear and was determined at the start of intervention﻿. |
| **Post-intervention bias** | **Authors’ judgement** | **Support for judgement** |
| Bias due to deviations from intended interventions | Low | All participants received intended intervention as described in surgical methods. |
| Bias due to missing data | Low (SFR)  Low (complications) | There were no missing data concerning the primary or secondary outcomes. |
| Bias in measurement of outcomes | Low (SFR)  Low (complications) | Blinding of assessors, ﻿personnel, or participants was not applied. However, we concluded with scientific judgement that the occurrence of events was unrelated to blinding. |
| Bias in selection of the reported results | Low (SFR)  Low (complications) | ﻿Although this is a retrospective study that is susceptible to selection bias of all reported results, the pre-specified outcomes—including both primary and secondary outcomes commonly reported in comparative endourology studies—parameters were all reported. |

| **Wen et al. 2016** | | |
| --- | --- | --- |
| **Bias** | **Authors’ judgement** | **Support for judgement** |
| Random sequence generation (selection bias) | Low | Quote: “67 qualified patients presenting with partial staghorn calculi were randomized to MPCNL group or ECIRS group with allocation ratio 1:1. The randomization for the patients was finished by statistical department of our hospital by computer-generated allocation sequence and opened to the operating surgeons before surgery.﻿” |
| Allocation concealment (selection bias) | Low | See above |
| Blinding of participants and operating room personnel (performance bias) | High | Blinding of urologist and operating room personnel is not feasible; judged as high risk of bias. |
| Blinding of non-operating room personnel (performance bias) | Low | Blinding of non-operating room personnel not addressed. However, we concluded with scientific judgement that the outcomes are unlikely to be influenced by a lack of blinding. |
| Blinding of outcome assessment (detection bias) | Unclear | Insufficient information to permit judgement |
| Incomplete outcome data (attrition bias) | Low (SFR)  Low (adverse events) | No missing outcome data on both SFR and adverse events/complications. |
| Selective reporting (reporting bias) | Low (SFR)  Low (adverse events) | Pre-specified (primary and secondary) outcomes are clearly published. |
| Other bias | Low | Grant funded |

| **Leng et al., 2018** | | |
| --- | --- | --- |
| **Pre-intervention bias** | **Authors’ judgement** | **Support for judgement** |
| Bias due to confounding | Serious | Adjustment was only made for stone characteristics. No adjustment was made for other ﻿clinicopathological factors. |
| Bias in selection of participants into the study | Unclear | This is a retrospective study comparing two surgical techniques (PCNL versus standard ECIRS). ECIRS may have been used in patients with a higher stone burden due to the complementary addition of retrograde ureteroscopy. Case selection may therefore have influenced the estimate of the treatment effect in the ECIRS arm. It was unclear how participants were selected for treatment with ECIRS over PCNL. |
| **At intervention** | **Authors’ judgement** | **Support for judgement** |
| Bias in classification of interventions | Low | Classification of interventions clear and was determined at the start of intervention﻿. |
| **Post-intervention bias** | **Authors’ judgement** | **Support for judgement** |
| Bias due to deviations from intended interventions | Low | All participants received intended intervention as described in surgical methods. |
| Bias due to missing data | Low (SFR)  Low (complications) | There were no missing data concerning the primary or secondary outcomes. |
| Bias in measurement of outcomes | Low (SFR)  Low (adverse events) | Blinding of assessors, ﻿personnel, or participants was not applied. However, we concluded with scientific judgement that the occurrence of events was unrelated to blinding. |
| Bias in selection of the reported results | Low (SFR)  Low (adverse events) | ﻿Although this is a retrospective study that is susceptible to selection bias of all reported results, the pre-specified outcomes—including both primary and secondary outcomes commonly reported in comparative endourology studies—parameters were all reported. |

| **Zhao et al. 2019** | | |
| --- | --- | --- |
| **Pre-intervention bias** | **Authors’ judgement** | **Support for judgement** |
| Bias due to confounding | Moderate | ﻿Important clinicopathological factors were appropriately balanced (sex, BMI, age, ASA grade); some minor differences in stone characteristics (size, burden) between the two arms. |
| Bias in selection of participants into the study | Moderate | Selection of patients may have been related to ECIRS and outcome, but the authors of the study used appropriate methods to adjust for selection bias.  Quote: ﻿"Seoul National University Renal Stone Complexity (S-ReSC) scoring system was used to classify and describe the complexity of renal calculi based on the number of sites involved in the renal pelvis, major or minor calyces [8].”  Quote: “Additionally, patients who were performed with preoperative treatment such as ESWL, FUS or PCNL, and supervened severe abnormal urinary anatomy were excluded, in case of influencing the actual results.” |
| **At intervention** | **Authors’ judgement** | **Support for judgement** |
| Bias in classification of interventions | Low | Classification of interventions clear and was determined at the start of intervention﻿. Each group received either ECIRS or mini-PCNL. |
| **Post-intervention bias** | **Authors’ judgement** | **Support for judgement** |
| Bias due to deviations from intended interventions | Low | All participants received intended intervention as described in surgical methods. |
| Bias due to missing data | Low (SFR)  Low (complications) | There were no missing data concerning the primary or secondary outcomes. |
| Bias in measurement of outcomes | Low (SFR)  Low (complications) | Blinding of assessors, ﻿personnel, or participants was not applied. However, we concluded on scientific judgement that the occurrence of events was unrelated to blinding. |
| Bias in selection of the reported results | Low (SFR)  Low (complications) | ﻿Although this is a retrospective study that is susceptible to selection bias of all reported results, the pre-specified outcomes including both primary and secondary outcomes parameters were all reported |

## **Appendix 4.** Characteristics of excluded studies with justifications for exclusion.

| Study | Reason for exclusion |
| --- | --- |
| Giusti 2018 | Technical report |
| Gómez-Regalado 2020 | Case report |
| Grande 2021 | Evaluated ECIRS as a treatment option for urinary tract carcinoma |
| Gücük 2013 | Study compared groups that underwent standard PCNL with a either a rigid or a flexible nephroscope |
| Hamamoto 2015 | Retrospective case series; no comparator group |
| Huang 2021 | Comparative analysis of multi-tract PCNL and single-tract ECIRS; excluded due to the use of multiple tracts in PCNL |
| Isac 2013 | ECIRS was not part of |
| Kang 2020 | Study evaluated endoscopic combined ultrasound-guided access (EUGA) with conventional ultrasound-guided access (UGA) for achieving renal access in ECIRS; both groups included ECIRS and only evaluated access methodology |
| Kuroda 2015 | Study that identified preoperative predictors for predicting success rate after ECIRS |
| Kwon 2017 | Study analyzes ECIRS in two arms. However, ECIRS was performed in both arms with the only variation being the side RIRS was performed, therefore not ECIRS versus PCNL/mini-PCNL |
| Manikandan 2016 | Retrospective case series on ECIRS with no comparator group |
| Serra 2012 | Retrospective case series (n = 21) on ECIRS with no comparator group |
| Taguchi 2021 | mini-ECIRS v. mini-ECIRS w/ ureteroscopy-assisted puncture |
| Tawfeek 2021 | Retrospective case series (n = 45) on ECIRS with no comparator group |
| Usui 2020 | Study comparatively analyzed mini-ECIRS versus standard ECIRS; excluded due to both groups including ECIRS |
| Yamashita 2017 | Retrospective case series (n = 75) evaluating stone size in patients who underwent ECIRS |
